# Subjective Confidence in the Response to Personality Questions: Some Insight Into the Construction of People’s Responses to Test Items

**DOI:** 10.3389/fpsyg.2020.01250

**Published:** 2020-06-24

**Authors:** Asher Koriat, Monika Undorf, Eryn Newman, Norbert Schwarz

**Affiliations:** ^1^Department of Psychology, Institute of Information Processing and Decision Making, University of Haifa, Haifa, Israel; ^2^Department of Psychology, School of Social Sciences, University of Mannheim, Mannheim, Germany; ^3^Research School of Psychology, The Australian National University, Canberra, ACT, Australia; ^4^Mind and Society Center, University of Southern California, Los Angeles, CA, United States,; ^5^Department of Psychology, University of Southern California, Los Angeles, CA, United States,; ^6^Marshall School of Business, University of Southern California, Los Angeles, CA, United States

**Keywords:** self-report measures of personality, consistency and variability, subjective confidence, State-Trait debate, response latency

## Abstract

Drawing on research on subjective confidence, we examined how the confidence and speed in responding to personality items track the consistency and variability in the response to the same items over repeated administrations. Participants (*N* = 57) responded to 132 personality items with a true/false response format. The items were presented five times over the course of two sessions. Consistent with the Self-Consistency Model, the confidence and speed with which an item was endorsed at its first presentation predicted the likelihood of repeating that response across the subsequent presentations of the item, thus tracking test-retest reliability. Confidence and speed also predicted the likelihood that others will make the same response, thus tracking inter-person consensus. However, confidence and speed varied more strongly with within-person consistency than with inter-person consensus, suggesting some reliance on idiosyncratic cues in response formation. These results mirror, in part, findings obtained in other domains such as general knowledge, social attitudes, and personal preferences, suggesting some similarity in the decision processes underlying the response to binary items: responses to personality items are not retrieved ready-made from memory but constructed at the time of testing, based on the sampling of a small number of cues from a larger population of cues associated with the item’s content. Because confidence is based on the consistency with which the cues support a response, it is prognostic of within-person consistency and cross-person consensus. Theoretical and methodological implications are discussed.

## Introduction

This article concerns the consistency and variability in people’s responses to self-report tests that are routinely used in the assessment of personality. The question of the stability in people’s responses and behavior has been the subject of heated debates for many years. The assessment of personality in terms of traits assumes that individual differences in the patterns of behavior associated with these traits are relatively stable across situations. This assumption has been challenged by evidence suggesting that the cross-situational consistency in behavior is low or moderate at best (May and Hartshorne, 1926; Mischel, 1968). At the same time, however, there is evidence that individual differences in the expressions of trait-related behavior are stable across time (e.g., Fleeson, 2004), and that trait measures predict many real-life variables (e.g., Paunonen, 2003).

In recent years, there has been increased effort to integrate between-person stability and within-person variability in an integrated conceptual framework (see Beckmann and Wood, 2017). Researchers proposed that the notion of traits needs to be modified to incorporate within-person variability. Fleeson (2001, 2004; see also Fleeson and Jayawickreme, 2015) argued that traits should be viewed as density distributions that represent how a person acts on different occasions. These distributions were obtained using an experience-sampling method in which participants reported on their thoughts, feelings, and behaviors on multiple occasions over time. Sun and Vazire (2019), who also used an experience-sampling methodology, reported results suggesting that people are aware of the fluctuations in personality that occur across different daily occasions. They showed that for some personality traits, peoples’ perceptions of their momentary states correlate with those of external observers, suggesting that people have insight into the fluctuations in their personality.

In turn, Baumeister and Tice (1988; see also Bem and Allen, 1974; Tellegen, 1988) proposed that traits should be conceived in analogy to attitudes. In the same way that not every person has an attitude about every issue, not all trait dimensions apply equally well to all people. Traits are said to constrain the behavior of traited persons, whereas untraited persons may take a broader range of trait-relevant actions. Therefore, focusing on traited individuals has the potential of increasing the consistency and predictive validity of measured traits (Kenrick and Stringfield, 1980).

Other researchers argued that within-person variability in trait levels is of interest in its own right. Fiske and Rice (1955) and Fleeson (2001) reported evidence suggesting that intra-individual response variability is reliably stable across time (see also Baird et al., 2006; Ferrando, 2011; Fleeson and Jayawickreme, 2015). Markus (1977), who focused on self-concept, argued that a well-articulated self-schema is essential for consistency in endorsements of personality items, and for observing a relationship between an individual’s responses to these items and his or her other judgments and actions.

The approach that we took in this study is different. We used several self-report scales that are intended to measure individual differences in personality traits. Focusing narrowly on the test-taking situation, we examined the consistency and variability in participants’ responses to these test items. We took advantage of a conceptualization that proved useful in tracking both the stable and variable contributions to the measurement of social beliefs and social attitudes (Koriat and Adiv, 2011, 2012; Koriat et al., 2016, 2018). Here, we wish to examine whether this general conceptualization can also apply to the assessment of personality traits.

Underlying this conceptualization is the assumption that when assessing personality traits on the basis of self-report responses, the participants themselves play a critical role as measurement instruments. Consider a participant who is presented with the following items: “I enjoy intellectual challenges”; “When I am confused about an important issue, I feel very upset.” What runs through the person’s mind in trying to decide whether to respond *yes* or *no* to these items? Possibly, the person recollects specific personal episodes, tries to recall what others think about him, compares himself to others, and so on (see Petty et al., 2007). Thus, even though the items involve one’s self-perception, people do not usually retrieve a ready-made response, but construct their response on the spot depending on the cues that are accessed at the time of responding. Indeed, proponents of the attitude-as-construction position have proposed that judgments about social attitudes are constructed online on the basis of the information accessible when making the judgment (Schwarz and Strack, 1985; Wilson and Hodges, 1992; Schwarz, 2007). A similar view was proposed with regard to people’s preferences (Lichtenstein and Slovic, 2006): it was argued that preferences are generally constructed in the process of elicitation. These views have emphasized the malleability and context-sensitivity of the responses to attitude and preference questions. We argue that the analysis of the cognitive processes involved in constructing a response to self-report measures of personality is of interest in its own right, and can also provide insight into the malleability inherent in the very assessment of personality traits. That analysis may also lead to a conceptualization in which response variability is conceived as an inherent property that must be taken into account in the assessment of personality (see Fleeson and Jayawickreme, 2015; DeMarree and Bobrowski, 2018).

Previous work, based on the Self-Consistency Model (Koriat, 2012), indicated that when people face two-alternative forced-choice (2AFC) items, the confidence and speed with which they endorse their response provide insight into the psychological processes underlying that response (Koriat, 2008, 2012). In particular, confidence and response speed can help track the consistency and variability in people’s responses to the same items. Results consistent with this assumption have been obtained for tasks tapping general knowledge and perceptual judgments. In this study, we examine whether this is also true for the responses to self-descriptive statements that are typically used in personality tests.

In what follows, we first review the Self-Consistency Model and the findings that support it and then apply the model to people’s responses to items that measure individual differences in personality.

## Choosing a Response To 2AFC Items: The Sampling Model

The Self-Consistency Model addresses the general question of how people choose between two options when they are presented with 2AFC items. In line with previous suggestions (Schwarz and Strack, 1985; Schwarz, 2007), it assumes that participants make their choice on the spot on the basis of a small number of cues that they retrieve. Specifically, they draw a sample of cues sequentially from a population of cues that is associated with that item, and evaluate the implication of each cue for the decision. The retrieval of cues is terminated either when a predetermined number of cues has been accessed or when several cues in a row have been found to favor the same decision (Audley, 1960). Once the retrieval of cues has terminated, the choice of a response is based on the balance of evidence in favor of the two alternative responses (see Vickers, 2001), and confidence is based on the consistency with which the response chosen is supported across the cues retrieved. Thus, subjective confidence is assumed to capture the reliability of the decision: it reflects essentially the assessed likelihood of making the same response in subsequent encounters with the item.

This rudimentary model (for details on the model and its relationship to other models, see Koriat, 2012) makes several predictions that have been largely confirmed across several different tasks (see Koriat and Adiv, 2016). We will review these predictions and findings and then examine their implications for the consistency and variability in the response to self-report measures of personality.

## Within-Individual Analyses

Consider the situation in which the same set of 2AFC questions is presented on different occasions. Some variability in the responses may be observed across different occasions (see Fiske and Rice, 1955), but confidence and response latency should be expected to track that variability. Specifically, the Self-Consistency Model predicts that participants should endorse their more frequent choice with greater confidence and speed than their less frequent choice (Koriat, 2012). For instance, a person who responds seven times to the item “Which sport activity would you prefer, (a) jogging, (b) swimming?” should be more confident in her frequent response (i.e., the response she chooses four times or more) than in her rare response (i.e., the response she chooses three times or less). In addition, the difference in confidence between participants’ more frequent and less frequent choices should increase with item consistency – the proportion of times that a more frequent response has been chosen across presentations. This means that a person’s confidence difference should be larger for items where she chooses her frequent response six times and her rare response once (high item consistency) than for items where she chooses her frequent response four times and her rare response three times (low item consistency). These predictions are based on the sampling assumption underlying the Self-Consistency Model: people sample their cues largely from the same pool of cues across occasions, although the specific set of cues retrieved may differ from one occasion to another. However, the choices that are associated with higher confidence are the more representative of the population of cues associated with the item.

These predictions have been confirmed in seven experiments in which participants were repeatedly presented with the same task (see Koriat et al., 2016). The tasks used included perceptual judgments, like-dislike judgments, social beliefs, social attitudes, and personal preferences, all involving 2AFC responses. For each task, the two responses to each item were classified for each participant as frequent or rare according to their relative frequencies across presentations. Consistency was defined for each item as the number of times the more frequent response was endorsed across presentations (between five and seven presentations depending on the experiment). All experiments yielded a pattern in which (a) mean confidence was higher for the frequently chosen response than for the rare response and (b) the frequent-rare difference in confidence tended to increase with cross-presentation consistency. The same general pattern was observed for response speed (see Figure 3 in Koriat et al., 2016). These results are consistent with the assumption that for each person, the aggregation of judgments across presentations discloses the characteristics of the item-specific population of cues from which the individual draws a sample of cues in each occasion (Koriat, 2012). These characteristics are largely responsible for the consistency in people’s responses to each item across occasions (Fleeson, 2004). Inconsistency stems from the fact that cognitive limitations constrain the number of cues that can be retrieved and consulted in each occasion, and that the response is based on the set of cues that are sampled at the time of making a judgment.

A second prediction follows from the assumption that confidence monitors the reliability of the response: confidence should be diagnostic of the replicability of the response across item repetitions. Indeed, in experiments in which the same task was repeated several times, the confidence and response speed with which a response was made in the first presentation of an item were found to predict the likelihood of making the same response in the subsequent presentations. This was true for perceptual judgments, social beliefs, social attitudes, personal preferences, and category membership decisions (Koriat, 2011, 2013; Koriat and Adiv, 2011, 2012; Koriat and Sorka, 2015).

These results bring to the fore the importance of considering both the stable and variable components in the assessment of self-report responses in many domains. Confidence and speed can help track the contributions of these components.

## Cross-Person Analyses: The Prototypical Majority Effect

Other predictions of the Self-Consistency Model concern cross-person consensus. An important assumption of the model is that in many domains, such as general knowledge, perceptual judgments, and social beliefs, people with the same experience largely share the item-specific population of cues from which they draw their sample in each occasion. This assumption is consistent with research on the wisdom of crowds, although that research has focused on tasks for which the response has a truth value (Surowiecki, 2004). The predictions from this assumption are referred to as the prototypical majority effect (PME, see Koriat et al., 2016, 2018): first, for each 2AFC item, the response made by the majority of participants should be endorsed with greater confidence, and shorter response latency than the minority response. Second, the majority-minority difference in confidence and response speed should increase as a function of the size of the majority – the proportion of participants who opt for the majority choice. This pattern of results is precisely what was found in research on social conformity (e.g., Festinger, 1954; Bassili, 2003; Petrocelli et al., 2007). However, Koriat et al. (2016, 2018) presented evidence that the PME pattern can occur independent of any social influence. Evidence for the predicted PME pattern was found for a number of domains, as reviewed by Koriat et al. (2016).

It should be stressed that the PME was observed both between-individuals and within-individuals: for each item, participants who chose the majority option tended to respond faster and with greater confidence than participants who chose the minority option. In addition, each individual tended to respond faster and with greater confidence when he/she chose the majority response than when he/she chose the minority response. The results of Koriat et al. (2018) further suggest that people are likely to opt for the majority choice and to endorse it with greater speed and confidence even when they have no idea what other people choose, and even when they are wrong in predicting the majority response.

## The Consensuality Principle: Some Constraints

The assumption that people with the same experience sample their cues largely from the same population of cues helped explain what was referred to as the consensuality principle (Koriat, 2008, 2011): confidence is correlated with the consensuality of the response – the likelihood that the same response will be made by the majority of other participants. Thus, for tasks for which the response has a truth value, confidence monitors the accuracy of the choice only because the consensual response tends to be the correct response. However, when consensuality and accuracy were disentangled by deliberately selecting items for which people tend to choose the wrong answer, the confidence-accuracy correlation was *negative*: wrong responses were endorsed with higher confidence than correct responses (Koriat, 2012, 2018). This was found to be the case across some 20 different tasks (see Koriat, 2018). In fact, confidence in the correctness of a response is more predictive of whether that response will be made by the majority of other participants than whether that response is correct or wrong (Koriat, 2019).

The consensuality principle is tenable for such domains as general knowledge and perceptual judgments because for these domains, people with similar experiences may be expected to share the same population of cues for each item by virtue of their adaptation to the same ecology (Juslin, 1994; Dhami et al., 2004). However, what happens in domains such as personality, for which stable differences exist between individuals? In these domains, we must assume that participants differ in the cues available to them and in the implications that they draw from these cues. Possibly, for such domains, confidence and response speed may prove to correlate more strongly with within-person consistency than with cross-person agreement. Indeed, such was found to be the case for social attitudes (Koriat and Adiv, 2011), social beliefs (Koriat and Adiv, 2012), and even more so for personal preferences (Koriat, 2013): when participants indicated their confidence in their response to a personal preference question (e.g., “do you prefer to use a pen or a pencil?”), confidence in the choice was predicted better from within-person agreement than from cross-person consensus: the partial *η*^2^, as an estimate of effect size, was 0.89 for within-person agreement, but only 0.14 for cross-person consensus.

## This Study

Because the processes underlying confidence judgments have been most extensively studied with 2AFC items, it is useful to rely on this format in a first exploration of the applicability of the Self-Consistency Model to responses to personality items. Many prominent personality tests, including the MMPI (Dahlstrom et al., 1972) and CPI (Gough, 1987), were developed with a 2AFC format, although other popular personality inventories (e.g., the Big Five measures) employ a graded response format. Accordingly, our participants made a true/false response to each 2AFC item and indicated their confidence in their response. Response latencies were also measured. Our intention was to see whether confidence and response speed provide insight into the process underlying the choice of a true/false response to self-descriptive items. Of key interest is whether confidence and response speed track the consistency and variability in people’s responses, similar to what had been found for items in other domains. To examine the predictions for within-person consistency, participants were presented with the same self-report questions five times.

In the within-individual analysis, we examined the prediction that the frequently made responses across repetitions should be associated with higher confidence and speed than the less frequently made responses. Results consistent with this prediction would suggest that responses to self-descriptive personality questions are also constructed on the spot on the basis of the cues that are sampled at the time of judgment. We also examined whether confidence and response speed monitor the replicability of responses: we tested the hypothesis that confidence in the first response to an item should predict the likelihood of repeating that response in the subsequent presentations of that item.

Turning next to cross-person consensus, the obvious prediction is that because of the idiosyncratic nature of the response to self-descriptive items, little relationship should be found between confidence and consensus. Two somewhat surprising observations, however, suggest that such might not be the case. First, the predictions concerning the relationship between social consensus and confidence were confirmed even for personal preferences (Koriat, 2013). Despite the idiosyncratic nature of everyday personal preferences, confidence and response speed yielded a clear PME, suggesting that the cues underlying choice and confidence are partly shared across people. A second observation comes from the study of social attitudes (Koriat and Adiv, 2011). The items used in that study measured roughly conservatism-liberalism views. When participants were divided into “conservatives” and “liberals,” both groups yielded a PME even when the responses were defined as majority or minority responses on the basis of the entire sample of participants. Clearly, participants at the opposite poles of an attitude dimension must sample their cues from overlapping but distinct distributions of cues, because otherwise they would not differ in their attitudinal judgments. Nevertheless, the results suggested that the cues shared across all participants contributed to people’s confidence in their choices.

Is it possible that even in the case of self-descriptive personality items, individuals draw on cues that are partly shared across people? If so, we should expect some evidence for a PME: confidence in a response should increase with the proportion of other participants who make that response.

Altogether, the approach underlying the present study is that even in the case of self-descriptions, people do not retrieve a ready-made response, but reach a decision on the basis of the cues that they access at the time of making the judgment (see also Baumeister and Tice, 1988). Therefore, the constructive, inferential process may yield some variability in the response across occasions, but confidence and response speed should track both the stability and variability across occasions.

In the study to be reported, we used four personality scales. Two scales have been assumed to correlate with the tendency to yield to conformity pressures. The other scales are generally unrelated to conformity. In order to test the predictions from the Self-Consistency Model, we used a 2AFC format for all items, and participants reported their confidence in the chosen response.

## Materials and Methods

The study received the approval of the University of Southern California University Park Institutional Review Board. All participants provided informed consent.

### Materials

The task included a total of 132 2AFC items from the following scales: (1) *Social Desirability* (SD). The SD scale of Crowne and Marlowe (1960) was used. It included 33 items. (2) *Fear of Negative Evaluation* (FNE). This scale was developed by Watson and Friend (1969). Here we used a brief version, which included 12 items (Leary, 1983). (3) *Need for Closure* (NFC). This scale (Webster and Kruglanski, 1994) included 47 items. (4) *Rational-Experiential Inventory* (RE) (Pacini and Epstein, 1999). The scale included 40 items. A true/false format was used for all the scales.

### Participants

Fifty-seven participants were recruited from the University of Southern California campus. They were asked to participate in a study on social beliefs and attitudes. After completing the two experimental sessions, they were paid $30.

### Procedure

The study consisted of five presentations (blocks) of the entire set of 132 items. These presentations were divided between two sessions so that Session 1 included two blocks, and Session 2 included three blocks. In each block, all items were administered. The two sessions took place on two separate days at least 2 days apart, and lasted about 1 h (Session 1) and 1–1.5 h (Session 2).

Participants were instructed to read statements concerning personal attitudes and traits, and to decide whether each statement was true or false as it pertains to them personally. They were instructed to click with the mouse the *True* or *False* boxes that appeared beneath each statement, and then to indicate their confidence on a scale from 0 to 100%; 0 means that they were completely unsure about their response, whereas 100 means that they felt absolutely confident about their response.

In each trial, each statement (e.g., “I hate to change my plans at the last minute”) was presented until participants pressed a *continue* box to indicate that they had finished reading it, at which time the response options true/false were added beneath the statement. Participants clicked one of the two response options. Response latency was measured, defined as the interval between the *continue* and the choice of a response. After clicking a *confirm* box, a confidence scale (0–100) was added beneath the alternative options, and participants marked their confidence by sliding a pointer on a slider using the mouse (a number in the range 0–100 corresponding to the location of the pointer on the slider was shown in a box). After clicking a second *confirm* box, the next trial began. Participants could change their response or their confidence judgment but not after clicking *confirm*.

The order of the items was random for each participant and block except that two practice items (different from one block to another) appeared at the beginning of each block. At the beginning of Blocks 2–5, participants were told that they would see the same items again, and that they should perform the same tasks on these items, as before.

## Results

In what follows, we first use the results across the five blocks to examine the predictions regarding the relationship between confidence and within-person consistency. We then turn to the effects of cross-person consensus, which will be examined using the results from Block 1 only.

Our primary method of analysis was Hierarchical Linear Modeling (HLM). This method has several advantages over repeated measures ANOVA because it includes the simultaneous estimation of within-subject and between-subjects variance (e.g., Quené and van den Bergh, 2004). Models were fit using the R packages lme4 and lmerTest (Bates et al., 2015; Kuznetsova et al., 2016). In all models, random intercepts for participants and items were specified. All categorical predictors were effect coded and all continuous predictors were centered at their mean. We report unstandardized regression coefficients. Specifying random intercepts for participants took into account that people differ reliably with respect to mean confidence judgments (Stankov and Crawford, 1997; Kleitman and Stankov, 2001). Thus, all results reported below were independent of people’s overall level of confidence.

**Figure 1:**
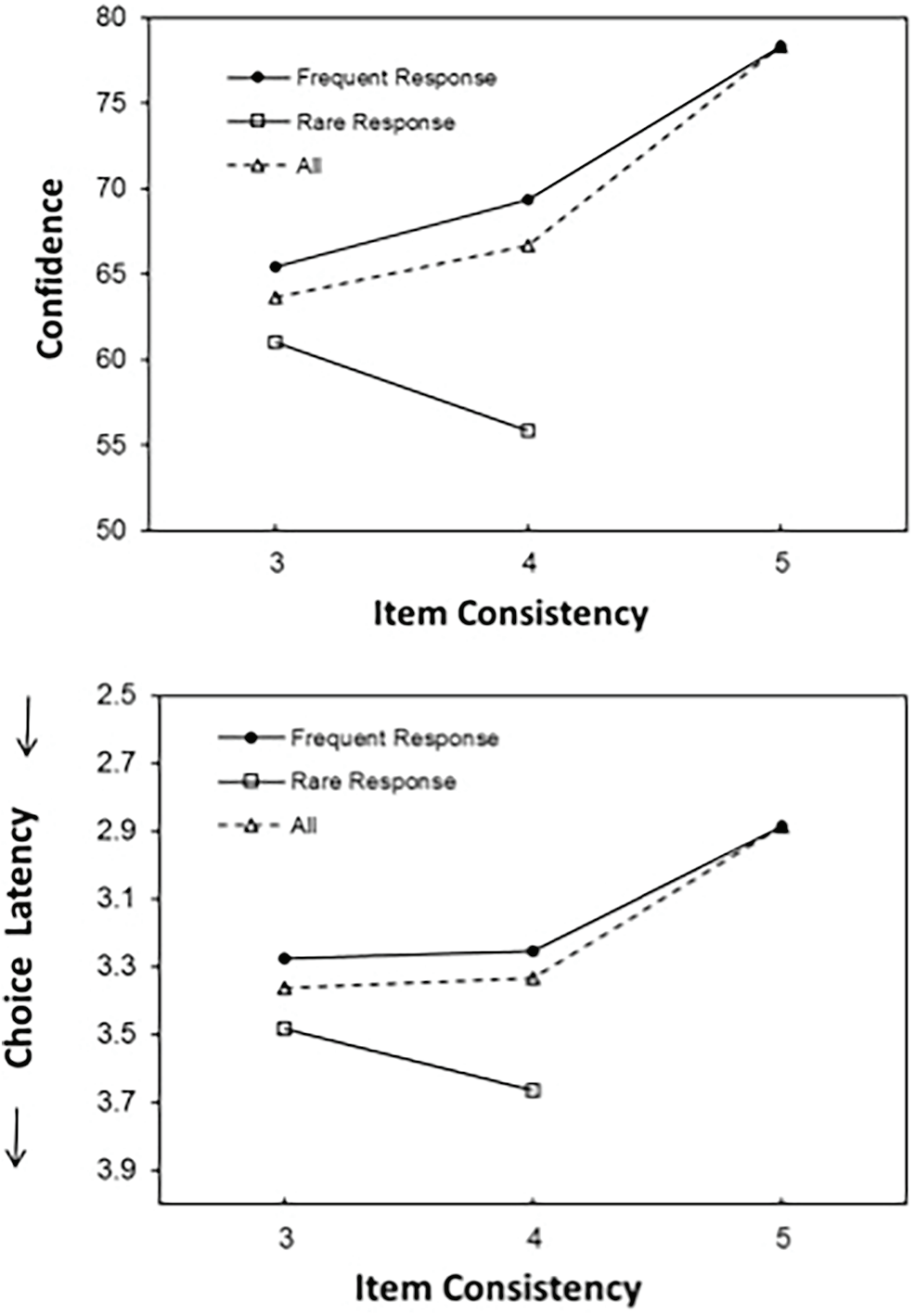
Mean confidence judgments (**top panel**) and response latency (**bottom panel**) for the frequent and rare responses as a function of item consistency – the number of times that the response was made across all five blocks.

**Figure 2:**
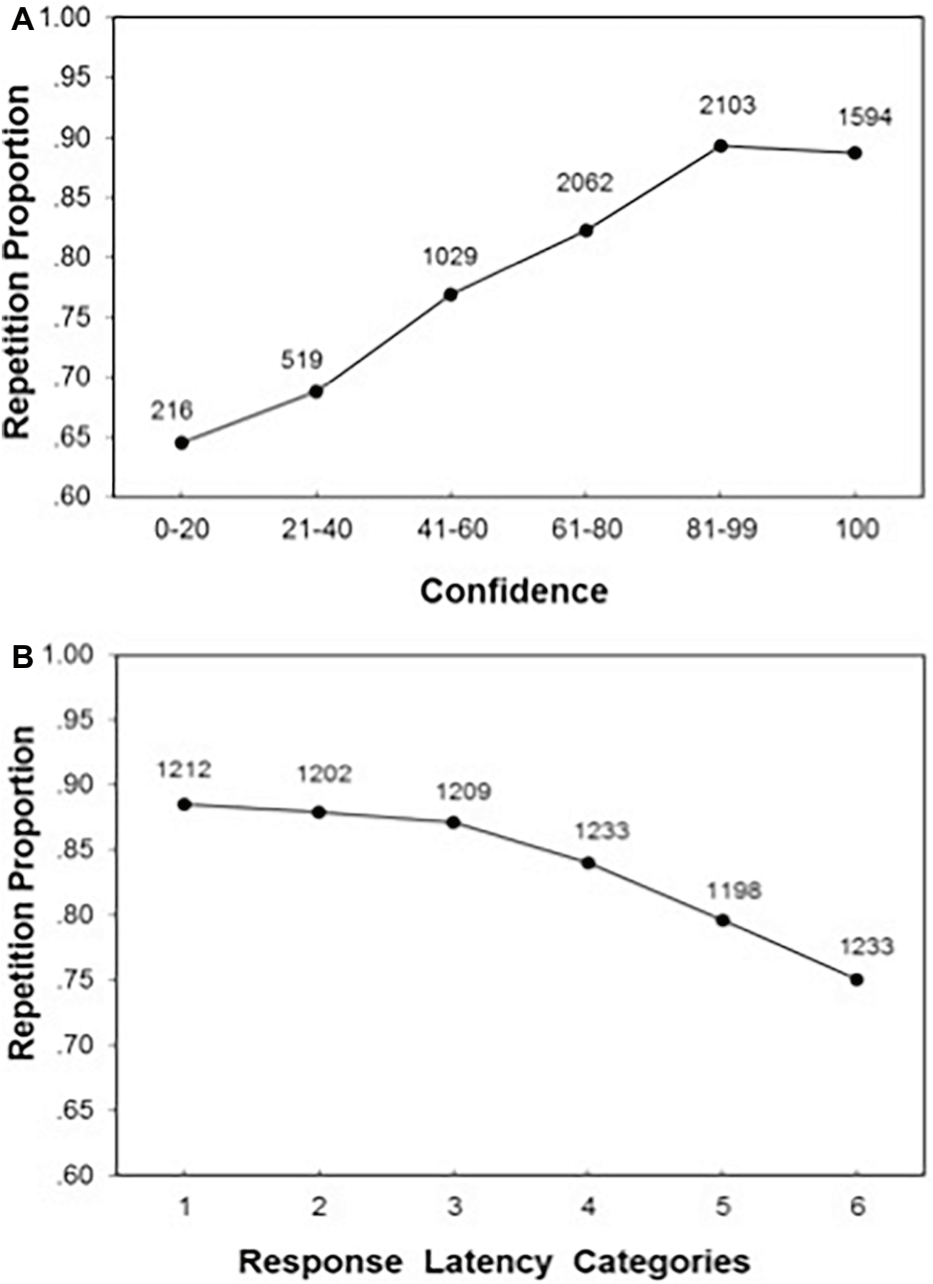
Panel **(A)** presents the likelihood of repeating the Block-1 response as a function of confidence in that response in Block 1. Indicated also is the number of observations in each confidence category. Panel **(B)** presents the same data for response latency.

### Within-Person Analyses

In general, participants tended to give the same response consistently throughout the five blocks, supporting within-person stability (see Fleeson, 2004). The likelihood of choosing the Block-1 response over the next four blocks averaged 0.83 across all participants.

#### Confidence as a Function of Response Consistency

For each participant, we classified all items into those that elicited the same response across all blocks (full consistency) and those exhibiting some degree of inconsistency (partial consistency). Confidence was significantly higher for the full-consistency items (*M* = 78.33, *SD* = 10.42) than for the partial-consistency items (*M* = 65.39, *SD* = 14.73), *t*(56) = 13.44, *p* < 0.0001, and *d* = 1.80. All 57 participants exhibited higher confidence for the full-consistency items than for the partial-consistency items, *p* < 0.0001, by a binomial test.

The previous analysis involved between-item effects on confidence. We turn next to between-response effects, comparing confidence for the participant’s frequent and rare responses. Figure 1 presents mean confidence for the two categories of items as a function of item consistency – the number of times that the frequent response was chosen across the five presentations. The figure also includes mean confidence for the full-consistency items. As expected, an HLM that predicted confidence from item consistency (ranging from 3 to 5) revealed that confidence increased with item consistency, *b* = 7.39 (*SE* = 0.15), *t*(37465) = 48.14, and *p* < 0.0001.

**Figure 3:**
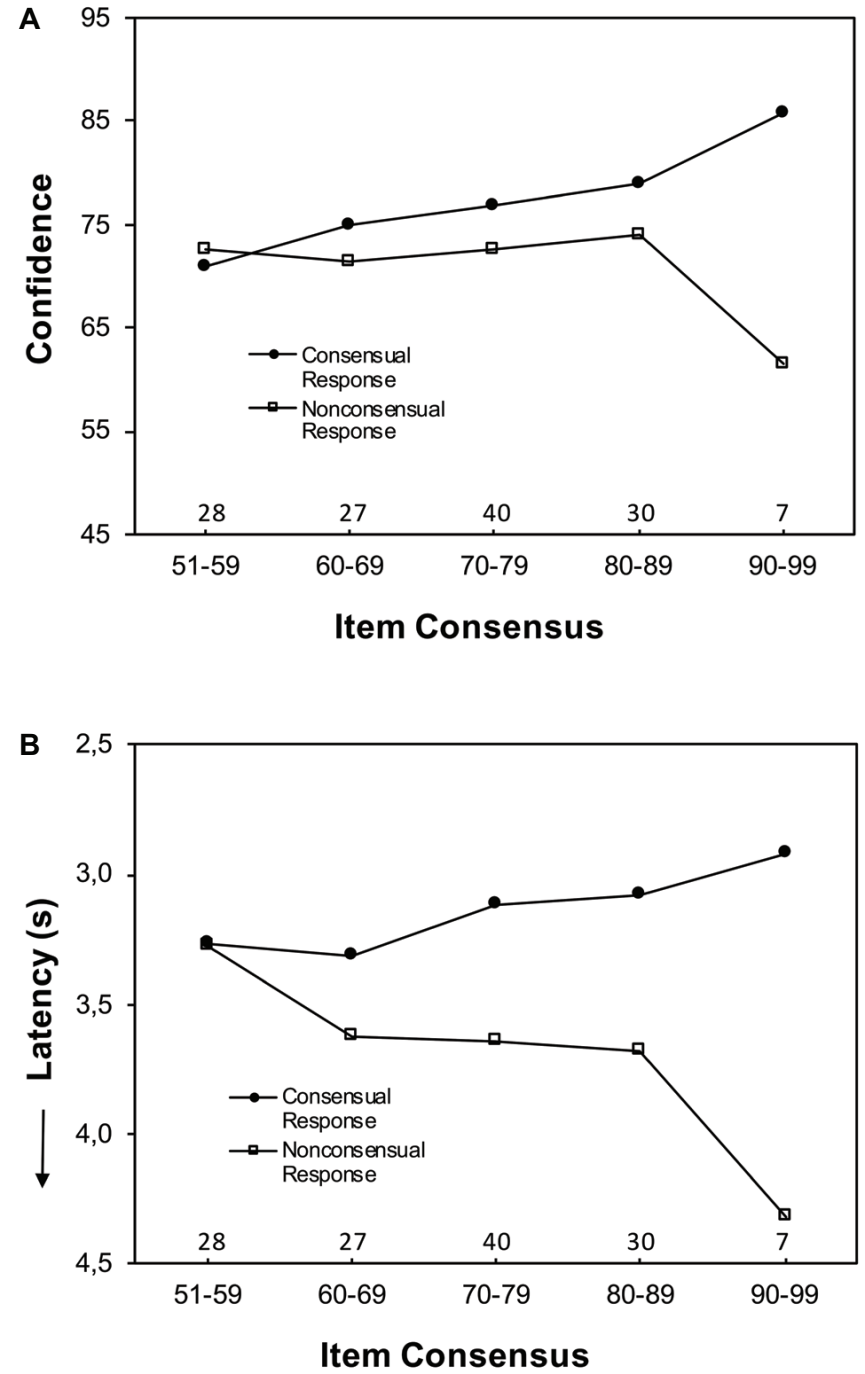
Mean confidence judgments Panel (**A**) and response latency Panel (**B**) in Block 1 for consensual and nonconsensual responses as a function of item consensus – the percentage of participants who made the majority response.

However, focusing on the partial-consistency items, confidence was significantly higher for the participants’ frequent responses (*M* = 67.74, *SD* = 14.78) than for the participants’ rare responses (*M* = 58.14, *SD* = 15.37), *t*(56) = 11.57, *p* < 0.0001, and *d* = 1.55. All participants exhibited the pattern of higher confidence for frequent than for rare responses, *p* < 0.0001, by a binomial test. Thus, participants were less confident when their response deviated from *their own* modal response. This pattern replicates results obtained for several other domains (Koriat et al., 2016) and is consistent with the Self-Consistency Model (Koriat, 2012).

As expected, the difference in confidence between the frequent and rare responses increased with item consistency. An HLM that predicted confidence from item consistency, response frequency (effect coded: −1 = rare, 1 = frequent), and their interaction yielded a main effect of response frequency, *b* = 10.22 (*SE* = 0.57), *t*(11197) = 17.82, and *p* < 0.0001, indicating higher confidence in frequent responses. A small but significant main effect of item consistency, *b* = −1.28 (*SE* = 0.49), *t*(11148) = 2.62, and *p* = 0.009, indicated that confidence decreased slightly with item consistency. However, the interaction was significant, *b* = 5.21 (*SE* = 0.47), *t*(11197) = 10.99, and *p* < 0.0001, indicating that the difference in confidence between the frequent and rare responses increased with item consistency. This pattern too replicates the pattern obtained for other tasks (Koriat et al., 2016).

#### Response Latency as a Function of Response Consistency

We turn next to the results for response latency. In all analyses of response latency, latencies below or above 2.5 SDs from each participant’s mean in each block were eliminated (3.14% for Block 1 and 2.99% across all five blocks)^1^. Consistent with previous results (Kelley and Lindsay, 1993; Koriat et al., 2006; Koriat, 2012), there was an inverse relationship between confidence and response latency. Thus, focusing on the results of Block 1, an HLM that predicted confidence from response latency indicated that confidence decreased significantly with response latency, *b* = −1.75 (*SE* = 0.12), *t*(7282) = 14.34, and *p* < 0.0001.

We examined the results for response speed using the same analysis as we did for confidence. The results, presented in Figure 1, largely mimic those for confidence. Response latency was significantly shorter for the full-consistency items (*M* = 2.88 s, *SD* = 0.74) than for the partial-consistency items (*M* = 3.33 s, *SD* = 0.90), *t*(56) = 12.34, *p* < 0.0001, and *d* = 1.65. This pattern was observed for 56 of the 57 participants, *p* < 0.0001, by a binomial test.

Focusing now on the partial-consistency items, an HLM predicted response latency from item consistency, response frequency, and their interaction. A main effect of response frequency, *b* = −0.18 (*SE* = 0.06), *t*(10710) = 3.04, and *p* = 0.002, indicated that response latency was shorter by 176 ms for the frequent responses than for the rare responses. However, neither the main effect of item consistency, *t* < 1, nor the interaction were significant, *b* = −0.06 (*SE* = 0.05), *t*(10710) = 1.20, *p* = 0.228. Thus, the difference in response latency between frequent and rare responses did not increase significantly with item consistency.

In sum, it is impressive that despite the high stability in participant’s responses across repetitions, the results yielded the typical pattern of higher confidence for the frequent than for the rare responses. For confidence, the difference between frequent and rare responses increased with item consistency.

#### Replicability of the Response as a Function of Confidence and Response Speed

Assuming that confidence and response speed monitor the reliability of the response, we would expect that the confidence and speed with which a response is endorsed in Block 1 should predict the likelihood of making that response in the subsequent presentations of the item. Figure 2A shows the likelihood of repeating the Block-1 response across the subsequent four blocks. For ease of exposition, the means are plotted separately for six categories of confidence judgments in Block 1. A logistic HLM showed that the likelihood of repeating the Block-1 response increased with people’s confidence in their Block-1 response, *b* = 0.03, *z* = 32.99, and *p* < 0.0001.

A similar pattern was observed for response latency (Figure 2B). The likelihood of repeating the Block-1 response across the subsequent blocks decreased significantly with people’s response latency in Block 1, *b* = −0.13, *z* = 16.46, and *p* < 0.0001. Thus, although participant exhibited some variability in their responses from one occasion to another, confidence and speed track the consistency of making the same response across occasions.

### Cross-Person Analyses

The results for the within-person consistency replicated closely those obtained for other tasks. However, we suspected that cross-person analyses may produce different results. The PME obtained so far was explained in terms of the assumption that populations of cues associated with a 2AFC question overlap in people with similar backgrounds. This assumption is less tenable for personality measures. We now examine the effects of cross-person consensus on confidence and response speed focusing only on the results from Block 1. We first determined the consensual response to each of the 132 items. Item consensus for each item was defined as the percentage of participants who chose the consensual response. It averaged 71.88% across items (range: 50.88–98.25%).

#### Confidence as a Function of Cross-Person Consensus

For ease of exposition, Figure 3A presents mean confidence judgments for consensual and nonconsensual responses for each of six item consensus categories (51–59, 60–69, 70–79, 80–89, 90–99, and 100%). Somewhat surprisingly, the results yielded a PME pattern similar to that found for other tasks such as general knowledge (Koriat, 2008) and perceptual judgments (Koriat, 2011). An HLM predicted confidence from response consensuality (effect coded: −1 = nonconsensual response, 1 = consensual response), item consensus, and their interaction. A main effect of response consensuality, *b* = 2.77 (*SE* = 0.28), *t*(7409) = 9.88, and *p* < 0.0001, indicated that participants were more confident when they made the response endorsed by the majority of participants (*M* = 76.15) than when they made the minority response (*M* = 72.08). A main effect of item consensus, *b* = 0.12 (*SE* = 0.03), *t*(198) = 3.68, and *p* < 0.001, indicated that confidence increased with increasing item consensus. A significant interaction, *b* = 0.18 (*SE* = 0.02), *t*(7057) = 7.71, and *p* < 0.0001, indicated that the impact of item consensus on confidence differed between consensual and nonconsensual responses. Separate HLMs for consensual and nonconsensual responses indicated that confidence in consensual responses increased with item consensus, *b* = 0.30 (*SE* = 0.03), *t*(129) = 9.02, and *p* < 0.0001, whereas confidence in nonconsensual responses decreased with item consensus, *b* = −0.12 (*SE* = 0.05), *t*(138) = 2.23, and *p* = 0.028.

#### Response Latency as a Function of Cross-Person Consensus

Similar analyses to those of confidence were applied to response latency. The results (for Block 1) are presented in Figure 3B. The pattern mimics largely the one obtained for confidence.

In an HLM predicting response latency, a main effect of consensuality, *b* = −0.19 (*SE* = 0.03), *t*(7167) = 6.98, and *p* < 0.0001, indicated that participants responded faster (*M* = 3.18 s) when they chose the consensual response than when they chose the nonconsensual response (*M* = 3.60 s). A significant interaction, *b* = −0.01 (*SE* = 0.002), *t*(6963) = 5.08, and *p* < 0.0001, indicated that the impact of item consensus on response latency differed between consensual and nonconsensual responses. The main effect of item consensus was not significant, *t*(190) = 0.50, and *p* = 0.615, and separate HLMs for consensual and nonconsensual responses indicated that response latency for consensual responses decreased with item consensus, *b* = −0.01 (*SE* = 0.003), *t*(132) = 3.34, and *p* = 0.001, whereas that for nonconsensual responses increased with item consensus, *b* = 0.01 (*SE* = 0.01), *t*(132) = 2.42, and *p* = 0.017.

In sum, despite the idiosyncratic nature of the responses to self-descriptive items, the results yielded a PME for both confidence and response speed as those found for other tasks.

#### Comparing the Effects of Response Consensus to Those of Response Consistency

We examined the possibility that the effect of consensus was relatively weak compared to that of within-person consistency. To do so, we predicted confidence and response latency from (a) consensus based on the responses made to an item in Block 1 by the remaining 56 participants, (b) consistency depending on the item’s within-participant frequency, and (c) their interaction. An HLM predicting confidence for Block 1 from consensus, consistency, and their interaction, yielded a main effect for consensus, *b* = 0.21 (*SE* = 0.03), *t*(133) = 7.76, and *p* < 0.0001, as well as for consistency, *b* = 6.67 (*SE* = 0.34), *t*(7492) = 19.64, and *p* < 0.0001. The interaction was not significant, *t*(7459) = 1.43 and *p* = 0.152. As suggested by the *t*-values, the effects of response consistency were stronger than those of response consensus. Indeed, the standardized regression weights amounted to 0.29 for consistency, and only to 0.01 for consensus. These results suggest that there was only a small overlap between the populations of cues from which participants sampled the cues underlying their choices and confidence.

We compared these results to those obtained for other domains. In a perceptual task that required comparing the length of two lines, the impact of response consistency on confidence was about the same in magnitude as the impact of response consensus (Koriat, 2011). In contrast, confidence in personal preferences such as whether one prefers to have a dog or a cat or to use a pen or a pencil, was impacted more strongly by response consistency than by response consensus (Koriat, 2013). These results seem to disclose the relative degree of cross-person communality in the populations of cues from which people draw their samples in responding to the items in each domain: the more idiosyncratic these populations of cues are, the weaker the impact of consensus relative to that of consistency.

A similar HLM for response latency yielded a main effect for consistency, *b* = 0.33 (*SE* = 0.03), *t*(7268) = 9.89, and *p* < 0.0001, a marginally significant main effect of consensus, *b* = 0.005 (*SE* = 0.002), *t*(133) = 1.90, and *p* = 0.060, and no interaction, *t*(7235) = 0.84 and *p* = 0.400. Thus, response latency varied with consistency, whereas consensus had only a marginal effect.

#### The Relationship Between Cross-Person Consensus and Within-Person Consistency

It may be expected that participants who exhibit a strong impact of self-consistency, suggesting greater reliance on idiosyncratic cues, would exhibit weaker impact of cross-person consensus. To examine this possibility, we calculated two indices for each individual. As an index of within-person consistency, we used the proportion of items for which each participant made the same response across all blocks. As an index of cross-person consensus, we used the proportion of items for which the participant’s response was the consensual response. Unlike what might have been expected, the correlation between the two indices was positive and significant, *r* = 0.46, *t*(55) = 3.81, and *p* < 0.001. Furthermore, an HLM predicting consensus in Block 1 from participants’ confidence in their Block-1 choice indicated that participants’ confidence predicted the likelihood that the same choice would be made by *other* participants, *b* = 0.07 (*SE* = 0.01), *t*(7524) = 11.80, and *p* < 0.0001 (see also Koriat, 2019). These results suggest that the shared core of cues that contributes to the effects of consensus on choice and confidence, also contributes to the impact of self-consistency.

#### The Effects of Consensus for Different Personality Scales

So far, we have analyzed the results across all the items included in this study, focusing on structural characteristics that follow from the sampling assumption underlying the Self-Consistency Model. We now examine how the results hold true across the personality scales used in this study.

As noted earlier, two of the scales—SD and FNE—have been assumed to correlate with the tendency to yield to conformity pressures (e.g., Marlowe and Crowne, 1961; Leary and Kowalski, 1997). We examined the possibility that the effects of consensus on confidence and response speed were mostly due to these scales. To do so, we compared the effects of consensus for the SD and FNE scales to those for the NFC and RE scales. Focusing on the results for Block 1, an HLM predicted confidence from consensus, scale type (effect coded: −1 = scales NFC and RE, 1 = scales SD and FNE), and their interaction. A main effect for consensus, *b* = 0.21 (*SE* = 0.04), *t*(131) = 6.03, and *p* < 0.0001, indicated that high consensus generally increased people’s confidence in personality items. A main effect of scale type, *b* = 0.72 (*SE* = 0.36), *t*(131) = 2.00, and *p* = 0.048, indicated higher confidence in responses to items that tap a tendency to yield to group pressure. Importantly, a non-significant interaction, *t*(131) = 1.86 and *p* = 0.066, indicated that the effect of consensus on confidence in personality items was not restricted to items that tap a tendency to yield to group pressure.

Response latency yielded a similar pattern of results. An HLM predicting latency from consensus, scale type, and their interaction, revealed a main effect of consensus, *b* = −0.009 (*SE* = 0.004), *t*(131) = 2.44, and *p* = 0.016, but no other significant effects, *t* < 1.

In sum, the effects of cross-participant consensus on confidence and response latency were not restricted to items that tap a tendency to yield to group pressure. Rather, these effects seem to be relatively independent of the content of the personality dimensions used.

## Discussion

The question of the consistency in people’s behavior is a central question in the study of personality and has important implications for personality assessment. Several researchers proposed that the notion of traits needs to be modified to incorporate within-person variability. For example, Fleeson (2001, 2004; see also Fleeson and Jayawickreme, 2015) argued that traits should be viewed as density distributions that represent how a person acts on different occasions. Other researchers suggested that inter-person differences in response variability are of interest in their own right (Fiske and Rice, 1955; Fleeson, 2001).

In this study, we focused narrowly on the stability and variability that can be observed in responses to items that are assumed to measure personality traits. We assumed that examination of the decision processes underlying people’s responses could shed light on the stability and variability in their responses. In our previous work, we successfully applied the Self-Consistency Model to 2AFC questions in different domains (Koriat, 2012; Koriat et al., 2016). Here, we examined which aspects of the model hold true for personality items. In what follows, we first summarize and discuss the results for the within-person analyses. We then turn to those concerning cross-person consensus. Finally, we note general implications of the results.

### Within-Person Consistency and Variability

The within-person analyses yielded a very similar pattern of results to that obtained in other domains, including perceptual decisions, social beliefs, social attitudes, personal preferences, and category membership decisions (see Koriat et al., 2016). This finding suggests that the process underlying the choice of a response to self-descriptive items has much in common with that underlying the response to 2AFC items in other domains. Specifically, the sampling assumption was found to provide insight into the processes that contribute to the consistency and variability in the response to personality test items. First, the results indicated that individual differences in responding to personality items are relatively stable across repeated test presentations (see Fleeson, 2004). Second, there was some variability in responding to test items across repeated presentations. However, frequent responses were endorsed with stronger confidence and shorter response latencies than rare responses, and the difference in confidence between the two types of responses increased with item consistency. Finally, the confidence and speed with which the response was made in the first presentation of an item predicted the likelihood of repeating that response in subsequent presentations of the item.

Taken together, the results are consistent with the idea that responses to self-descriptive items are not retrieved ready-made from memory but are constructed online at the time of responding to the items. Online construction is assumed to entail the sampling of a small number of cues from the larger population of cues associated with the item. Across occasions, people sample their cues from the same population of cues, resulting in generally consistent responses across repeated item presentations. However, the samples may differ because of random factors or differences in context or mood, resulting in some variability across occasions. As a result, in a minority of occasions, participants may opt for a response that departs from *their own* typical response, but their confidence in that response will be relatively low. Thus, the confidence and speed with which a response is endorsed capture both the stable, replicable components of the decision, and the variability that can ensue from the sampling process.

These results have implications for test construction. They suggest that the confidence and response speed with which responses are made can help item selection when the goal is to increase the test-retest reliability of the scale as a whole. Of course, exclusive selection of items associated with high confidence and speed may not be functional when the interest is also to track individual differences in response consistency and variability (see below).

### The Impact of Cross-Person Consensus

Turning next to the results for cross-participant consensus, these results were somewhat surprising. Despite the idiosyncratic nature of the responses to personality items, people responded relatively faster and more confidently when endorsing the response given by the majority of participants. Confidence also yielded the typical PME: the difference between consensual and nonconsensual responses increased with increasing item consensus.

Possibly, despite the idiosyncratic nature of personality measures, test items tend to elicit cues that overlap across participants. As noted earlier, in a study of social attitudes (Koriat and Adiv, 2011) that used items measuring conservatism-liberalism views, a PME emerged for both “conservatives” and “liberals” even when the responses were classified as majority or minority on the basis of the entire sample of participants. In addition, cues differ in the ease with which they come to mind. Indeed, results suggest that differences in the familiarity and accessibility of cues have systematic effects on confidence independent of the content of these cues (Koriat, 2008).

However, the impact of consensus on confidence and response speed was much more limited than that of response consistency, suggesting that the populations of cues underlying personality-related items are largely idiosyncratic. Nevertheless, the results suggest a shared core of cues that underlies the response to personality test items, and this core impacts the confidence and response speed with which that response is endorsed.

### Some General Implications

What are the general implications of the results of this study? The Self-Consistency Model was initially applied to tasks for which the binary response has a truth value: general knowledge and perceptual judgments. In these tasks, the confidence in the response conveys the person’s degree of conviction that the answer chosen is the *correct* answer. However, what is the meaning of subjective confidence in the context of such tasks as social attitudes, personal preferences, or category-membership judgments, for which the response does not have a truth value? Koriat (2012, 2018) argued that in all of these domains, including those for which the response has a truth value, subjective confidence actually monitors the reliability of the response: it represents an assessment of the likelihood that a new sample of cues drawn from the same population will support the same response.

The results of the present study suggest that this is also the meaning of subjective confidence in the response to a personality statement: it reflects the assessment that the response is “reliable.” What is notable is that across several domains, confidence and response latency were found indeed to predict the replicability of the response (see Koriat et al., 2016), and this was also true for the personality items used in this study. Thus, both repeated testing and confidence judgments can help identify the stable, replicable components in people’s responses to personality tests.

Whereas the focus of this study was on confidence in the response to a particular item, research suggests that overall confidence in the responses to a particular scale is also diagnostic of the reliability of the of scale score. Shoots-Reinhard et al. (2015) used several individual-differences measures that are related to political behavior. They found that people who endorsed their responses with overall higher confidence evidenced higher stability in their scores over time. Test scores associated with higher confidence were also more predictive of political outcomes than those associated with lower confidence.

It is of interest to relate our results to the idea of the wisdom of the inner crowd (Vul and Pashler, 2008; Herzog and Hertwig, 2009, 2014; Hourihan and Benjamin, 2010; Litvinova, 2020). This idea was applied to tasks for which the response has a truth-value. For these tasks, it was found that when a person provides several judgments, the aggregated judgment tends to be closer to the truth than any of the individual judgments. According to the Self-Consistency Model (Koriat, 2018), this is because in many domains, the reliable judgment is also the correct judgment. In general, however, the aggregation of individual responses across different occasions provides a clue to the central tendency of the population of cues from which the person samples the cues in each occasion. It is this tendency that is responsible for the consistency of responses across occasions.

Indeed, several researchers have called for a repeated assessment of personality across different occasions. As noted earlier, Fleeson (2001, 2004; see also Fleeson and Jayawickreme, 2015) proposed to view traits as density distributions. These distributions can be measured by the frequency with which a particular trait is expressed in different occasions (see also Sun and Vazire, 2019). It may be argued that the sampling of cues assumed to underlie test-takers’ responses to personality items constitutes in part a mental simulation of the procedure used by Fleeson (2004) and Sun and Vazire (2019). For example, in responding to the item “Do you have difficulty controlling your feelings,” respondents might scan their memory for relevant personal episodes in deciding between *yes* and *no*. If so, confidence judgments may provide a rough estimate of the variability that would be expected across different occasions. Clearly, however, more research is needed to examine whether the confidence and response latency obtained in a single administration of test items can capture part of the consistency and variability with which people express trait-related behavior under real-life conditions.

Another question concerns the proposal that within-person variability constitutes a stable dimension of individual differences that is worthy of investigation in its own right (Fiske and Rice, 1955; Fleeson, 2001; Baird et al., 2006; Ferrando, 2011). This proposal was made in particular in connection of the notion of Self-Concept Clarity (Baumgardner, 1990; Campbell, 1990; DeMarree and Bobrowski, 2018). As noted earlier, Markus (1977) argued that a well-articulated self-schema is essential for consistency in endorsements of personality items. It is of interest to examine whether the methodology used in the present study can be applied to examine the possibility of reliable individual differences in the consistency and clarity of self-descriptions.

Finally, our results suggest differences between domains in the relative impact of consensus versus consistency. It might be interesting to examine whether there are also reliable individual differences in this respect. Although the impact of consensus and that of consistency were found to correlate positively across participants, differences between individuals may also convey information about the extent to which the cues underlying a person’s responses to personality and self-concept questions are idiosyncratic.

## Data Availability Statement

The datasets generated for this study are available on request to the corresponding author.

## Ethics Statement

The studies involving human participants were reviewed and approved by University of Southern California University Park Institutional Review Board. The patients/participants provided their written informed consent to participate in this study.

## Author Contributions

Conception and design of the study: AK and NS. Data acquisition: EN. Analysis: MU and AK. Writing and editing: AK, MU, EN, and NS.

### Conflict of Interest

The authors declare that the research was conducted in the absence of any commercial or financial relationships that could be construed as a potential conflict of interest.

## Footnotes

^1^None of our conclusions changed when analyzing log-transformed response latencies.
